# Suicide reduction in Canada during the COVID-19 pandemic: lessons informing national prevention strategies for suicide reduction

**DOI:** 10.1177/01410768211043186

**Published:** 2021-09-22

**Authors:** Roger S McIntyre, Leanna MW Lui, Joshua D Rosenblat, Roger Ho, Hartej Gill, Rodrigo B Mansur, Kayla Teopiz, Yuhua Liao, Ciyong Lu, Mehala Subramaniapillai, Flora Nasri, Yena Lee

**Affiliations:** 1 Mood Disorder Psychopharmacology Unit, University Health Network, University of Toronto, Toronto, ON M5T 2S8, Canada; 2 Institute of Medical Science, University of Toronto, Toronto, ON M5S 1A8, Canada; 3 Department of Psychiatry, University of Toronto, Toronto, ON M5S 1A8, Canada; 4 Department of Pharmacology, University of Toronto, Toronto, ON M5S 1A8, Canada; 5 Department of Psychological Medicine, Yong Loo Lin School of Medicine, National University of Singapore, 10 medical Dr, Singapore, 117597, Singapore; 6 Institute for Health Innovation and Technology, National University of Singapore, 21 Lower Kent Ridge Rd, Singapore 119077, Singapore; 7 Department of Medical Statistics and Epidemiology, School of Public Health, Sun Yat-sen University, 135 Xingang W Rd, Bin Jiang Lu, Haizhu Qu, Guangzhou Shi, Guangdong Sheng, China; 8 Department of Psychiatry, Shenzhen Nanshan Center for Chronic Disease Control, 7 Huaming Road, Nanshan, Shenzhen City, Guangdong Province, China

**Keywords:** COVID-19, pandemic, suicide, unemployment, public health, mental health, depression, bipolar disorder

## Abstract

**Objective:**

The objective of this research was to evaluate the impact of federal, public health and social support programs on national suicide rates in Canada.

**Design:**

Cross-sectional study.

**Setting:**

Canadian National Database (i.e., Statistics Canada) and Statista.

**Participants:**

Population-level data, and economic and consumer market data.

**Main Outcome Measures:**

Suicide mortality data, population data and unemployment data were obtained from available statistical databases (e.g. Statistics Canada). We quantified suicide rate by dividing the total number of suicide deaths by the national population expressed as a rate per 100,000 population.

**Results:**

Overall suicide mortality rate decreased in Canada from 10.82 deaths per 100,000 in the March 2019 - February 2020 period to 7.34 per 100,000 (i.e. absolute difference of 1300 deaths) in the March 2020 - February 2021 period. The overall Canadian unemployment rate changed from an average monthly rate of 5.7% in 2019 to 9.5% in 2020.

**Conclusion:**

Our results indicate that for the first post-pandemic interval evaluated (i.e., March 2020 - February 2021), suicide rates in Canada decreased against a background of extraordinary public health measures intended to mitigate community spread of COVID-19. An externality of public health measures was a significant rise in national unemployment rates in population measures of distress. Our results suggest that government interventions that broadly aim to reduce measures of insecurity (i.e., economic, housing, health), and timely psychiatric services, should be prioritised as part of a national suicide reduction strategy, not only during but after termination of the COVID-19 pandemic.

## Introduction

The global COVID-19 public health crisis, the population-based interventions to mitigate COVID-19 transmission (e.g. social distancing, shelter-in-place edicts) and the financial/employment insecurity as a consequence of COVID-19 are an unprecedented risk to mental health and wellbeing.^1^ The global mental health crisis can be conceptualised as a syndemic insofar as a global loneliness pandemic was a prelude, and likely exacerbated in some countries, by COVID-19. Many of the mental health risks of loneliness are also ascribed to COVID-19 (e.g. suicidality, increased risk of depression).^2^ In keeping with this view, surveys conducted in high-, as well as low- and middle-income countries, during the COVID-19 pandemic have documented increased rates of distress as well as an increase in intimate partner violence, and mental illness emergency service utilisation during COVID-19.^3–6^

Unexpected, abrupt and large changes in economic/employment security have been reported to be associated with higher rates of completed suicide during previous economic crises.^7–9^ The association between indices of macroeconomic distress and increase in suicide is, however, complicated by the multifactorial causes of suicide as well as potential mitigating factors including government interventions that allocate resources to mental health services, vocational retraining and economic security (e.g. wage subsidy, debt forbearance).^10,11^

During the first five months of the COVID-19 pandemic, suicide rates in Japan were reported to have decreased.^12^ This initial decrease in suicide rates was immediately followed by a significant increase in suicide exceeding baseline pre-COVID-19 rates, particularly in women and youth (i.e. under 20 years of age).^12^ It is possible that government assistance during the first few months (which was later discontinued) of the pandemic in Japan partially mitigated suicide risk.^12^

The reduction in financial provisions by the Japanese government was temporally associated with a significant rise in suicide in Japan for the first time in 30 years.^12^ The differential increase of suicide in young women is hypothesised to be due to increased economic insecurity, decreased social cohesion/engagement, as well as exposure to domestic abuse and intimate partner violence.

The government of Canada responded to COVID-19 with a stringent shutdown of the economy and a surfeit of provisions. For example, the Canadian Emergency Response Benefit provided financial support to employed and self-employed Canadians of $2000 (CAD) every 4 weeks for up to 28 weeks.^13^ In addition, the Canadian Emergency Student Benefit provided $1250 (CAD) every 4 weeks for a maximum of 16 weeks.^14^ Moreover, recommendations were made by the Canadian Mortgage and Housing Corporation, a Crown Corporation of the Government of Canada, to allow for mortgage forbearance.

Along with federal programmes, provincial and municipal governments across Canada increased funding for emergency childcare.^15^ Collectively, the foregoing social programmes were in addition to the deployment of funding for emergency psychiatric services in the form of access to 24/7 crisis lines as well as the provision of psychotherapeutic and counselling services at no charge to Canadian residents.^16,17^

As a consequence of the COVID-19 pandemic, Canada, like most other countries, also recorded a significant increase in unemployment. Historically, unemployment and economic downturn have been associated with an increase in suicide rates which has been projected in both the United States and Canada as a consequence of COVID-19 unless mitigation measures were taken.^18,19^

Economies in Europe post-Great Recession that divested significant government resource to persons affected by unemployment/economic security demonstrated significant mitigation of suicide risk at the population level.^9^ The ample social programmes put in place by the Federal Government of Canada against a background of high and abrupt unemployment provides an opportunity to evaluate the mitigating effects of social/financial provisions on suicide levels at a national level.

Herein, we report on suicide rates in Canada during the first 12 months following the World Health Organization declaration of the global pandemic. The 12-month timeframe was chosen as data were available from Statistics Canada. In addition, the 12-month timeframe between March 2020 and February 2021 provides the opportunity for cross-national comparison with Japan wherein a biphasic outcome has been reported coinciding with the provision and termination of social benefits also against a background of decreased employment.

## Materials and methods

### Data sources

Annual, national-level suicide mortality data and population data were obtained from Statistics Canada.^20,21^ The population data represent values recorded by Statistics Canada from the fourth quarter (i.e. October to December). Statistics Canada is a federal statistics agency that captures economic-, societal- and environmental-related data on behalf of the federal government of Canada.^22^ Unemployment data were obtained from Statista, which is an online database that provides economic and consumer market data.^23^ The Vital Statistics – Death Database records the number of deaths attributed to suicide based on an administrative survey quantifying deaths among Canadian residents and non-residents. Suicide mortality was codified using the International Statistical Classification of Diseases and Related Health Problems, 10th revision (ICD-10) codes for intentional self-harm (i.e. X60-X84, Y87.0).

Statistics Canada data were publicly available and did not require ethics approval. Suicide mortality data for 2020–2021 are classified as provisional as they do not reflect all deaths that occurred during this time as a result of reporting delays.

### Statistical methods

We quantified suicide rate by dividing the total number of suicide deaths by the national population expressed as a rate per 100,000 population. Suicide count was quantified by summing the number of suicide deaths per week in each month.

## Results

**Figure 1.**
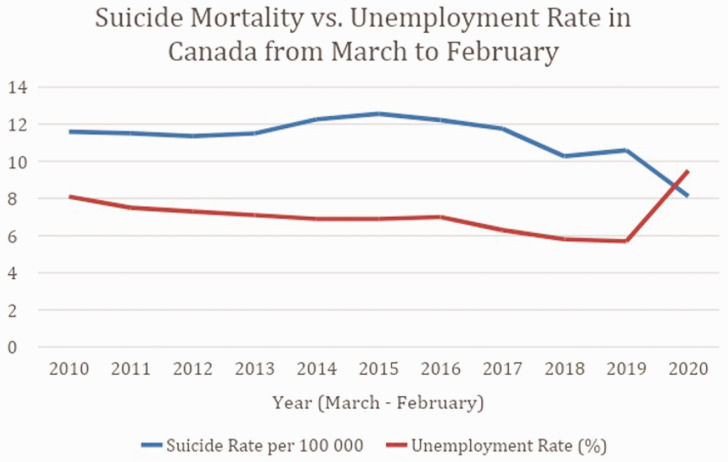
Annual unemployment rate and suicide mortality from March to February in Canada from 2010 to 2021.

Annual suicide rates and unemployment from 2010 to 2021 are displayed in Figure 1 and summarised in Table 1. Overall suicide mortality rate decreased in Canada from 10.82 deaths per 100,000 in March 2019 - February 2020 (i.e. 4090 suicide deaths in a population of 37,802,043) to 7.34 per 100,000 (i.e. 2790 deaths in a population of 38,008,005) in March 2020 - February 2021 (i.e. absolute difference of 1300 suicide deaths). The Canadian unemployment rate overall changed from an average monthly rate of 5.7% in 2019 to 9.5% in 2020.

**Table 1.** Total population, annual suicide rate and annual unemployment rate from March to February in Canada from 2010 to 2021.

Time period	Total population	Suicide rate per 100,000	Unemployment rate
March 2010 – February 2011	34,131,683	11.7046675	8.1
March 2011 – February 2012	34,457,998	11.4487208	7.5
March 2012 – February 2013	34,836,008	11.3388423	7.3
March 2013 – February 2014	35,211,866	11.5160043	7.1
March 2014 – February 2015	35,559,047	12.3034793	6.9
March 2015 – February 2016	35,822,894	12.6734596	6.9
March 2016 – February 2017	36,258,726	12.1350099	7.0
March 2017 – February 2018	36,721,242	11.6962275	6.3
March 2018 – February 2019	37,249,240	10.2686659	5.8
March 2019 – February 2020	37,802,043	10.8195211	5.7
March 2020 – February 2021	38,008,005	7.34055892	9.5

## Discussion

Herein, we observed a decrease in suicide completion across Canada during the first year of the COVID-19 pandemic. The Canadian suicide rate per 100,000 during the decade prior to 2020 has been relatively consistent, providing a reliable benchmark rate for comparison. The overall decrease in the rate of suicide during the first year of the pandemic equates to approximately 1300 fewer deaths relative to March 2019 - February 2020.

The decrease in overall suicide rates in Canada was contemporaneous with significant increases in unemployment. In keeping with the view that macroeconomic indices are associated with suicide, it was projected that in Canada and the United States, there would be an increase in completed suicide (i.e. ‘deaths of despair’) unless financial provisions and social programmes, as well as other services (e.g. mental health programmes) were immediately initiated to specifically address aspects of insecurity (e.g. economic, housing).^18,19^

The results observed 12 months after the start of the pandemic in Canada (i.e. decreased suicide rates) cohere with results reported in other countries wherein government provisions were immediately enacted.^24–26^ It has also been observed that the rate of suicide has not increased in China during the early stages of COVID-19, which is hypothesised to be due to an emphasis placed on community and family support mechanisms.^27,28^

Japan, which historically has high suicide rates, reported a steady decrease in cross-national suicide rates up until 2020.^12^ Immediately following the onset of the global COVID-19 pandemic, the Japanese federal government shut down the economy, including the closure of schools followed by social financial provisions.^12^ Between February and June 2020, the suicide rate in Japan decreased by 14%. The discontinuation of financial provision was immediately followed by a significant increase in suicide especially in young women.^12^

It is noteworthy that the observed decrease in the Canadian suicide rate alongside an increase in rates of psychological distress, mental illness and reports of suicidality reflects the multifactorial and discrete phenomenology of suicide during the COVID-19 pandemic.^29–32^ The results observed herein indirectly substantiate the multifactorial aspects related to population risk and resiliency as it relates to suicide.^29^ The results herein also underscore the importance of evaluating suicide rates separately from rates of distress and mental illness in both clinical and epidemiologic samples.

For example, in Canada, rates of mental distress and associated functional impairment increased during COVID-19.^33^ It is a testable hypothesis that the psychiatric first-aid services that were initiated by the government of Canada along with the social programmes mitigated suicide risk. An explanatory framework may also implicate aspects of collective resiliency as a societal factor mitigating suicide in the general population during times of a population-level stressor.^34^

Limitations of our data are the relatively brief window of 12 months. It cannot be assumed that the trajectory of suicide would continue at its current trend, nor can it be assumed that the labour market dynamics, macroeconomic indicators (e.g. inflation), consumer sentiment, government provisions and/or access to psychiatric care will remain static.

Between 2000 and 2018, the United States recorded not only a significant increase in suicide, but also an increase in death due to unspecified falls and unintentional poisonings. It is possible that the overall suicide rate in the United States may be greater than what is reported if it is assumed that some of the increase in unspecified and unintentional causes represent misclassified suicides.^29^ Comparing March 2019 - February 2020, to March 2020 - February 2021 data in the Statistics Canada database, there did not appear to be a cross-national trend for increase in unspecified and unintentional causes of death.^20^ The possibility that the total number of suicides is not accurately captured is suggested by evidence indicating decreased emergency room visits overall for psychiatric emergencies and, specifically, suicidality.^35^

Suicide data released by Statistics Canada are reported separately from accidental/unintentional poisonings, as well as alcohol-induced mortality cases.^36^ Statistics Canada reported an increase in unintentional poisoning in 2020 (e.g. Alberta and Ontario).^36^ The British Columbia Coroners Serve also observed an increase in illicit drug toxicity-related deaths in 2020 versus 2019.^36^ Among individuals aged 0 to 44, and 45 to 64, there was a significant increase in accidental poisonings observed in 2020 when compared to 2019 and 2017 (which was the height of the opioid crisis). A similar upward trajectory was also reported for alcohol-induced mortality among individuals aged 0 to 44, as well as 45 to 64, when compared to 2019. Taken together, suicide deaths reported for March 2020 - February 2021 are likely to underestimate the total number of deaths as an indirect consequence of the COVID-19 pandemic.

Furthermore, the results from our analysis should be interpreted with caution as the data capturing suicide deaths for 2020–2021 period are provisional. Provisional data for the 2020 reference year are incomplete (i.e., 93% complete) as deaths due to suicide require lengthy investigation by coroners or medical examiners.^36^ Consequently, the data herein are likely to underrepresent the true value of deaths attributed to suicide.

A further limitation is that we do not have age- and/or gender-specific data. We also do not have data as they relate to ethnic and racial minorities or indigenous communities, which are known to be differentially affected by inequity in health in Canada and elsewhere.^37^

## Conclusion

Our results indicate that for the first post-pandemic interval evaluated, suicide rates in Canada decreased against a background of extraordinary public health measures. A national imperative in Canada (and globally) should be to reduce suicide rates.^38,39^ Our results suggest that government interventions that broadly aim to reduce measures of insecurity (i.e., economic, housing, health), and provide timely psychiatric services, should be prioritised as part of a national suicide reduction strategy, not only during but after termination of the COVID-19 pandemic.

## Supplemental Material

sj-pdf-1-jrs-10.1177_01410768211043186 - Supplemental material for Suicide reduction in Canada during the COVID-19 pandemic: lessons informing national prevention strategies for suicide reduction Click here for additional data file. Supplemental material, sj-pdf-1-jrs-10.1177_01410768211043186 for Suicide reduction in Canada during the COVID-19 pandemic: lessons informing national prevention strategies for suicide reduction by Roger S McIntyre, Leanna MW Lui, Joshua D Rosenblat, Roger Ho, Hartej Gill, Rodrigo B Mansur, Kayla Teopiz, Yuhua Liao, Ciyong Lu, Mehala Subramaniapillai, Flora Nasri and Yena Lee in Journal of the Royal Society of Medicine

## Declarations

**Competing Interests:** Leanna M.W. Lui is a contractor to Braxia Scientific Corp. Yena Lee is the Chief Research Officer of Braxia Scientific Corp. Dr. Roger McIntyre has received research grant support from CIHR/GACD/Chinese National Natural Research Foundation; speaker/consultation fees from Lundbeck, Janssen, Purdue, Pfizer, Otsuka, Takeda, Neurocrine, Sunovion, Bausch Health, Novo Nordisk, Kris, Sanofi, Eisai, Intra-Cellular, NewBridge Pharmaceuticals, Abbvie. Dr. Roger McIntyre is a CEO of Braxia Scientific Corp. Kayla M. Teopiz has received personal fees from Braxia Scientific Corp. Dr. Joshua D Rosenblat has received research grant support from the Canadian Institute of Health Research (CIHR), Canadian Cancer Society, Canadian Psychiatric Association, Academic Scholars Award, American Psychiatric Association, American Society of Psychopharmacology, University of Toronto, University Health Network Centre for Mental Health, Joseph M. West Family Memorial Fund and Timeposters Fellowship and industry funding for speaker/consultation/research fees from Janssen, Allergan, Lundbeck, Sunovion and COMPASS. He is the Chief Medical and Scientific Officer of Braxia Scientific and the medical director of a Braxia Health.

**Ethics approval:** Not applicable.

**Guarantor:** RSM.

**Contributorship:** Authors contributed equally.
