# Supplementary material for: Suicide reduction in Canada during the COVID-19 pandemic: lessons informing national prevention strategies for suicide reduction
Source: J R Soc Med. 2021 Sep 22;114(10):473–9. doi: 10.1177/01410768211043186 (PMC8532219; doi:10.1177/01410768211043186)
Supplement: sj-pdf-1-jrs-10.1177_01410768211043186 - Supplemental material for Suicide reduction in Canada during the COVID-19 pandemic: lessons informing national prevention strategies for suicide reduction [file sj-pdf-1-jrs-10.1177_01410768211043186.pdf]

Disclosure statement

RSM has received research grant support from Global Alliance for Chronic Diseases/Canadian Institutes of Health Research (CIHR)/National Natural Science Foundation of China's Mental Health Team Grant; speaker/consultation fees from Lundbeck, Janssen, Purdue, Pfizer, Otsuka, Takeda, Neurocrine, Sunovion, Bausch Health, Novo Nordisk, Kris, Sanofi, Eisai, Intra-Cellular, NewBridge Pharmaceuticals, Abbvie. Dr. Roger McIntyre is a CEO of Braxia Scientific Corp.

LMWL is a contractor to Braxia Scientific Corp.

JDR is the medical director of the Braxia Health (formally known as the Canadian Rapid Treatment Center of Excellence and is a fully owned subsidiary of Braxia Scientific Corp) which provides ketamine and esketamine treatment for depression; he has received research grant support from the American Psychiatric Association, the American Society of Psychopharmacology, the Canadian Cancer Society, the Canadian Psychiatric Association, the Joseph M. West Family Memorial Fund, the Timeposters Fellowship, the University Health Network Centre for Mental Health, and the University of Toronto and speaking, consultation, or research fees from Allergan, COMPASS, Janssen, Lundbeck, and Sunovion.

RBM has received research grant support from PSI Foundation.

KMT has received personal fees from Braxia Scientific Corp.

YL has received personal fees from Braxia Scientific Corp.

All other authors declare no conflict of interest and/or financial disclosures.
